# Blood parameters in neonatal foal and colostrum quality as possible early markers for increased risk of developing *Rhodococcus equi* pneumonia

**DOI:** 10.3389/fvets.2025.1654052

**Published:** 2025-08-29

**Authors:** María Villalba-Orero, Camila A. Gómez, Marta Valero-Gónzalez, Noelia Venegas, Guadalupe Criado, María Martín-Cuervo

**Affiliations:** ^1^Department of Animal Medicine and Surgery, Faculty of Veterinary Medicine, Universidad Complutense de Madrid, Madrid, Spain; ^2^Complutense Veterinary Teaching Hospital, Universidad Complutense de Madrid, Madrid, Spain; ^3^Grupo de Investigación Medicina y Cirugía Animal (MECIAN), Animal Medicine Department, Faculty of Veterinary, University of Extremadura, Cáceres, Spain; ^4^Private Equine Practitioner, Barcelona, Spain

**Keywords:** *Rhodococcus equi*, foal, pneumonia, Brix, biochemistry, creatinine, gamma GT

## Abstract

**Background:**

*Rhodococcus equi* is a facultative intracellular bacterium recognized to cause pneumonia in foals aged 1 to 6 months. Currently, it remains a challenge to identify foals at risk.

**Hypothesis/Objectives:**

We hypothesize that a certain grade of immaturity may enhance the risk for future *R. equi* infection. This study aims to analyze blood parameters and passive immunity transfer within the first 24 hours of life as predictive markers for the development of *R. equi* pneumonia during the first 6 months of life.

**Methods:**

A total of 207 Arabian or Arabian-crossed breed foals from the same breeding center, from birth to 6 months of age, were included. Blood samples were obtained from foals during the first 24 hours after birth. Parameters analyzed were hematocrit (Hto), total white blood cell count (WBC), total plasma proteins (PT), albumin (ALB), fibrinogen (FBG), urea (U), creatinine (CREA), gamma-glutamyl transferase (GGT), iron (Fe), and serum immunoglobulin G (IgG). In addition, colostrum Brix was measured. Foals were classified into the following groups: foals presenting *R. equi* (R group) and healthy foals (H group), which showed no signs of pneumonia during a surveillance period of six months.

**Results:**

Comparisons were performed between the two groups, and univariable and multivariable logistic regression were used to assess possible predictors for *R. equi* development. Of the 207 foals, 25 became ill (12.08 %). Foals with *R. equi* showed lower U levels [H: 29 (23-37) Vs R: 24 (20-31); *p* = 0.04], as higher CREA [H: 1.3 (1.1-1.6) Vs R: 1.5 (1.3-1.7); *p* = 0.03], and GGT [H: 14 (10-24) Vs R: 21 (12-39); *p* = 0.004] than foals with a healthy status. Multivariable logistic regression highlighted that higher GGT at birth was associated with *R. equi* development.

**Discussion:**

High values of GGT and CREA have been associated with fetal immaturity, which could be related to immaturity of the immune system, especially of alveolar macrophages, and may predispose to early infection by *R. equi*. Interestingly, neonatal GGT may serve as a possible risk factor for developing the infection.

## 1 Introduction

*Rhodococcus equi* (*R. equi*) is a Gram-positive bacterium recognized for causing pneumonia in foals, representing a common cause of economic losses in the equine industry (1). As a facultative intracellular bacterium, it relies on its ability to survive within macrophages (2). This phenomenon is associated with the presence of a plasmid encoding virulence genes, among which vapA is considered the most crucial (3–5). The expression of the virulence-associated protein A (VapA) is markedly enhanced under iron-restricted conditions (6). Foals commonly develop *R. equi* between 1 and 6 months of age. However, exposure to this pathogen has been reported during the first week of life (7, 8). Enhanced intracellular replication of *R. equi* in bronchoalveolar macrophages of young foals, accompanied by age- and macrophage-specific cytokine expression patterns, may contribute to the pathogen’s pulmonary tropism and the age-dependent susceptibility to infection (9).

The primary manifestation of *R. equi* infection is a respiratory disorder, characterized by the development of pyogranulomatous lesions in the lungs (10). The first clinical signs may include sporadic or intermittent cough, pyrexia, lethargy, respiratory distress, reduced appetite, and weight loss (11). However, extrapulmonary infections and immunomediated inflammatory disorders are also common (12–15). Diagnosis typically relies on clinical presentation, hematological abnormalities, as well as radiographic and ultrasonographic evidence of pulmonary lesions (16). Additionally, cytological evidence of intracellular Gram-positive or acid-fast coccobacilli and/or detection of virulent *R. equi* in tracheal aspirates can be pursued (17, 18).

Several reports agree that the primary risk factor for pneumonia is the concentration of virulent *R. equi* in the environment (19–21) and the foal’s exposure to this pathogen (22, 23). Differences in the host’s innate and adaptive immune responses (24) and genetic variations (25) are also considered significant factors contributing to the disease’s development. Despite considerable advances in understanding this pathogen, controlling and preventing *R. equi* pneumonia in equine farms remains a challenge. Consequently, many efforts are focused on elucidating predictors of the disease before its clinical onset (26–28). Ultrasound diagnosis has been used as a screening method to identify affected animals early on (29), although it has been found that this diagnostic method is more useful for assessing the severity of the disease than as a screening tool (30). Something similar happens with radiographic diagnosis, where its most evident utility is in locating deep abscesses or determining the prognosis in affected animals (31). Acute-phase inflammation proteins have been explored as predictive indicators of the disease, as well as the significance of leukocyte count (27, 32, 33). However, their relationship with other blood parameters as predisposing factors is still unknown.

This study aims to assess whether hematological and biochemical blood parameter levels, as well as the colostrum quality in newborn foals, may anticipate the subsequent development of *R. equi* pneumonia. These parameters could potentially serve as biomarkers for the early detection of the infection.

## 2 Materials and methods

### 2.1 Animals

This study included all foals born healthy in a breeding farm of Arabian and Anglo-Arabian equine barn in 2018, located in the Autonomous Community of Catalonia (Spain). Foals were considered healthy at birth when their vital signs and hematology were within the physiological ranges for their age, exhibited normal behavior, and had adequate total IgG concentrations. All foals were under surveillance from birth to 6 months of age, both by farm workers and the veterinarians employed in the center. The diagnosis of *R. equi* was based on clinical signs and confirmed by PCR.

### 2.2 Sample collection

Before foals were 24 h old, venous blood samples were collected in tubes with EDTA anticoagulant and lithium heparin from the jugular vein to perform hematological and biochemical analysis, respectively. Hematological analyses and colostrum quality assessments were performed at the time of sample collection. For the measurement of biochemical parameters, the plasma was separated and frozen. Samples were frozen at −80°C and transferred to the Clinical Laboratory of the Veterinary Hospital of the University of Extremadura, where they were subsequently processed.

### 2.3 Hematology and serum biochemistry

The hematological analysis was performed with Mindray BC-5300 equipment, parameters measured were hematocrit (Hto; %) and total white blood cell count (WBC; ×10^8^/μL) specifying the count for lymphocytes, neutrophils, and monocytes. The biochemical analysis was performed with Crony instruments Saturno 100 equipment, and total plasma proteins (TP; g/dL), albumin (ALB, g/dL), fibrinogen (FBG, g/dL), urea (U, mg/dL) creatinine (mg/dL), gamma-glutamyl transferase (gamma GT; U/L); iron (Fe, ng/dL), and immunoglobulin G (IGG; mg/dL) was measured by quantitative determination, using MAI Animal Health’s DVM Rapid Test™ II.

### 2.4 Brix

Additionally, right after the delivery, the colostrum produced by the corresponding mothers was assessed using a Brix scale refractometer. The quality of the colostrum was measured within the first 12 h postpartum.

### 2.5 Diagnosis of *Rhodococcus equi* pneumonia

The foals included in this study were kept under supervision for their first 6 months of life and were checked every week from 3 weeks of age using an Easote MyLab™OneVET ultrasonography equipment equipped with the SV3513 VET rectal linear 10–5 Mhz-probe. The presumptive diagnosis of *R. equi* pneumonia was made based on the detection of clinical signs, such as cough, runny nose, pyrexia, lethargy, respiratory distress, decreased appetite, and weight loss, together with the detection of pulmonary abscesses compatible with pyogranulomatous pneumonia on the ultrasound examination. The diagnosis was confirmed by PCR. Accordingly, foals were classified into the following groups: foals presenting *R. equi* (R group) and healthy foals (H group), which showed no signs of pneumonia during this surveillance period.

### 2.6 Statistical analysis

Normality distribution was assessed using the Kolmogorov–Smirnov test. Data are presented as means ± standard deviations (SD) for variables with a normal distribution, and as the medians with interquartile ranges (IQT; 25th–75th) when normality could not be assumed. Firstly, data were grouped in foals that developed *R. equi* during the first 6 months of life (R group) or not (H group), and a Student *T*-test or Mann–Whitney *U* test (parametric or non-parametric, respectively) was applied to determine differences in measured parameters. The development of a subgroup in the R group with survivors and non-survivors was not performed, as only 2 foals died. In addition to establishing a relationship between the studied parameters at born and the outcome of development *R. equi*, all variables obtaining a *p* < 0.20 in the comparative analysis performed between R and H groups (neutrophils, albumin, urea, creatinine, gamma GT, and IGG) were selected for the logistic regression. Univariable logistic regression was performed to establish crude univariate odds ratios (OR) and 95% confidence intervals (CI) and a multivariate analysis was done to finally calculate the risk factor that better fix the model. The multivariate logistic regression model was built using automatic backward stepwise selection. Statistical differences were considered if *p* < 0.05. All statistical analyses were performed using SPSS statistical software (IBM SPSS Statistics 20; Chicago, IL, United States) and graphics were performed using GraphPad Prism version 9 (San Diego, CA, United States).

## 3 Results

### 3.1 Patient population

This retrospective study involved a total of 207 Arabian or Arabian-crossed breed foals. Of the 207 foals included, 25 foals (12.08%) developed pneumonia due to *R. equi* during the first 6 months of life (R group), and the remaining 178 foals maintained healthy conditions (H group).

### 3.2 General outcome

Twenty-two sick foals developed abscesses more than 1 cm in diameter and received rifampicin (10 mg/kg every 12 h) and azithromycin (10 mg/kg every 24 h) until the resolution of clinical signs. The other 3 foals showed abscesses less than 1 cm in diameter and did not receive antibiotics (additional information in Supplementary Table 1). The survival rate of the studied population was 92% (23/25) in the R group and 100% (178) in the H group.

### 3.3 Comparative analysis

Corresponding demographic data and blood work parameters in H and R groups are described in Table 1. Comparing H and R group, no statistical differences were observed for Hto (H: 39.7 ± 4.0 vs. R: 40 ± 3.2), total WBC (H: 7.663 ± 2.430 vs. R: 7.228 ± 2.470), and its components, total plasma proteins [H: 6.0 (5.3–6.7) vs. 6.0 (5.5–6.5)], albumin [H: 3.3 (3.1–3.5) vs. 3.4 (3.2–3.5)], fibrinogen [H: 378 (361–408) vs. 378 (327–463)], iron (H: 268.8 ± 89.6 vs. R: 255.2 ± 60.8), IGG levels (H: 1322.35 ± 539.187 vs. R: 1491.64 ± 657.577) at birth.

**Table 1:** Comparison of hematological data recruited at born, of 178 healthy foals (H group) and 25 foals that develop *R. equi* (N = 212 foals).

Parameters		H group		R group	*p*-value
	*n*	Mean ± SD or median (25th–75th)	*n*		
Hematocrit (%)	178	39.7 ± 4.0	24	40 ± 3.2	0.562
White blood cells (x10^8^/μL)	177	7.663 ± 2.430	25	7.228 ± 2.470	0.404
Neutrophils (×10^8^/μL)	176	4.870 ± 1.738	25	4.294 ± 1.516	0.117
Lymphocytes (×10^8^/μL)	178	1.975 (1.040–3.210)	25	2.250 (1.230–3.690)	0.672
Monocytes (×10^8^/μL)	178	0.315 (0.220–0.410)	25	0.320 (0.180–0.460)	0.881
Total plasma proteins (g/dL)	147	6.0 (5.3–6.7)	24	6.0 (5.5–6.5)	0.661
Albumin (g/dL)	149	3.3 (3.1–3.5)	24	3.4 (3.2–3.5)	0.133
Fibrinogen (g/dL)	12	378 (361–408)	21	378 (327–463)	0.667
Urea (mg/dL)	102	29 (23–37)	25	24 (20–31)	**0.045**
Creatinine (mg/dL)	102	1.3 (1.1–1.6)	25	1.5 (1.3–1.7)	**0.027**
Gamm GT (U/L)	94	14 (10–24)	24	21 (12–39)	**0.004**
Iron (ng/dL)	101	268.8 ± 89.6	24	255.2 ± 60.8	0.693
IGG (mg/dL)	178	1322.35 ± 539.187	25	1491.64 ± 657.577	0.155
Colostrum	158	0.272 (0.240–0.300)	24	0.260 (0.240–0.300)	0.854

Quantitative normally and non-normally distributed data are expressed as mean ± standard deviation (SD) or median (25th/75th percentile), respectively. Values are considered statistically significant with a *p* < 0.05 (indicating in bold font). Student *t*-test was used for quantitative normally distributed data and Mann–Whitney *U* test for quantitative non-normally distributed data.

Gamma GT, gamma-glutamyl transferase; n, number of data included for each analysis; 25th–75th, interquartile ranges.

**Figure 1:**
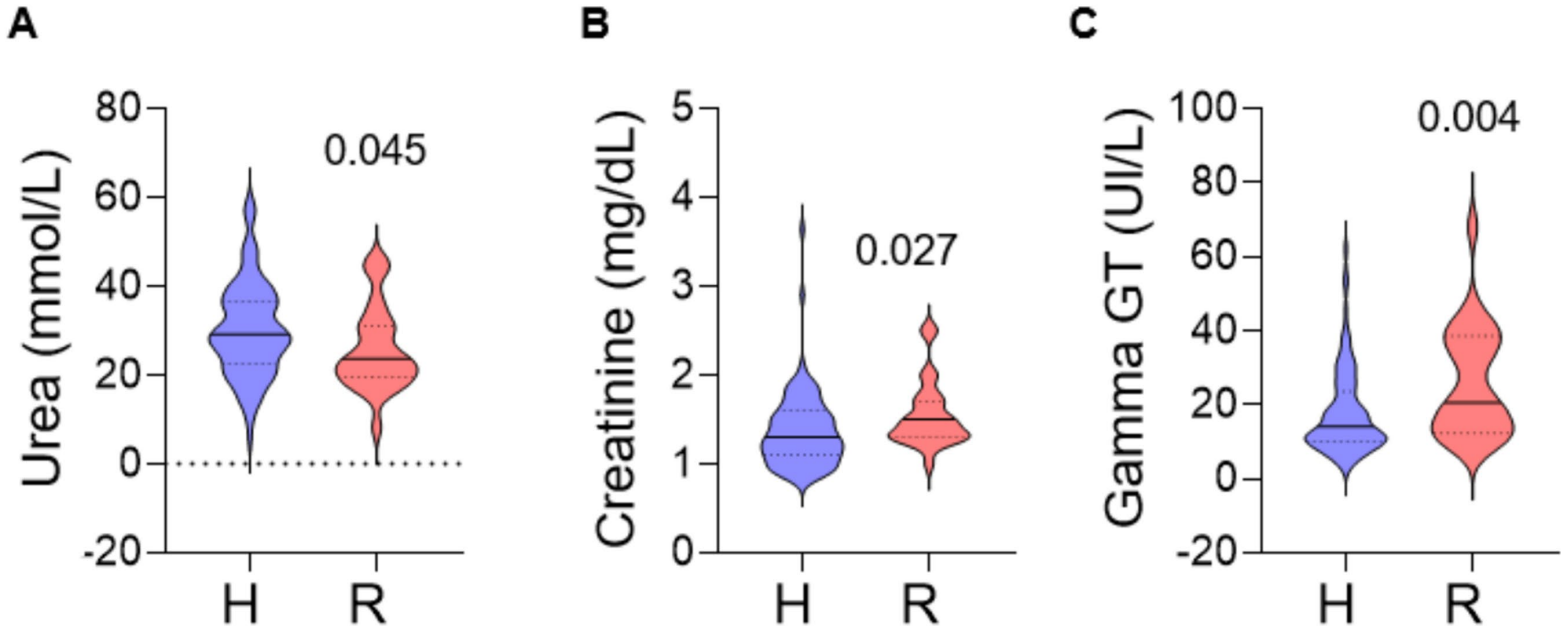
Violin plots representing a full distribution of raw data highlighting median (central line), and interquartile range (lower and upper lines). Comparisons are performed for blood urea **(A)**, creatinine **(B)**, and gamma GT **(C)** between foals that developed *Rhodococcus equi* in the first 6 months of life and those that remain healthy. Samples were collected during the first 24 h after birth. H, healthy group; R, *Rhodococcus equi* group.

However, as represented in Figure 1, foals with *R. equi* showed lower urea [H: 29 (23–37) vs. R: 24 (20–31); *p* = 0.04], as well as higher creatinine [H: 1.3 (1.1–1.6) vs. R: 1.5 (1.3–1.7); *p* = 0.03], and gamma GT [H: 14 (10–24) vs. R: 21 (12–39); *p* = 0.004] than foals with a healthy status. The two foals that died presented an urea of 44 and 19 (mmol/L), a creatinine of 2, 5, and 1 (mg/dL) and a gamma GT of 46 and 20 (Ul/L).

### 3.4 Brix

As shown in Table 1, 189 colostrum samples were evaluated, of which 24 were produced by the mothers of foals that subsequently developed *R. equi* pneumonia. Comparison between H and R groups highlighted that there was no significant difference regarding the quality of colostrum suckled and colostrum [H: 0.272 (0.240–0.300) vs. 0.260 (0.240–0.300)]. There was also no significant difference in IgG concentration between these 2 groups.

### 3.5 Factors associated with the development of *Rhodococcus equi*

Association between measurements of variables in foals born with the future development of *R. equi* was studied, including neutrophils, albumin, urea, creatinine, gamma GT, and IGG (variables obtaining a *p* < 0.20 in the comparative analysis performed between R and H groups, Table 1). As shown in Table 2, the univariable logistic regression evidenced that neutrophil count, albumin, urea, creatinine, and IGG at birth were not independently associated with future *R. equi*. However, this analysis reveals that higher blood levels of gamma GT at born are a risk factor for the development of the disease (OR: 1.05; 95% IC: 1.01–1.08; *p* = 0.008) and that higher urea levels tend to increase the risk of *R. equi* (OR: 0.96; 95% IC: 0.91–1.00; *p* = 0.068). For the multivariable logistic regression, a total of 115 foals (55.3%; 92 and 23 foals in the H and R groups, respectively) were automatically included. Variables that finally were maintained in the regression were gamma GT (OR: 1.043; 95% IC: 1.01–1.01; *p* = 0.019) and urea (OR: 0.956; 95% IC: 0.91–1.01; *p* = 0.070). These, formally studied parameters, presenting higher gamma GT at birth, were associated with *R. equi* development.

**Table 2:** Logistic regression showing data analyzed to establish risk factors associated with the development of *R. equi* of 178 healthy foals (H group) and 25 foals (*N* = 212 foals).

Parameters	*R. equi* development			
	Univariable analysis		Multivariable analysis	
	OR (95% CI)	*p*-value	Adjusted OR (95% CI)	*p-value*
Neutrophils (×10^8^/μL)	0.82 (0.64–1.05)	0.118		
Albumin (g/dL)	1.51 (0.5–4.60)	0.467		
Urea (mg/dL)	0.96 (0.91–1.00)	0.068	0.96 (0.91–1.01)	0.070
Creatinine (mg/dL)	1.12 (0.68–1.84)	0.648		
Gamma GT (U/L)	1.05 (1.01–1.08)	**0.008**	1.04 (1.01–1.01)	**0.019**
IGG (mg/dL)	1.00 (1.00–1.00)	0.157		

Gamma GT, gamma-glutamyl transferase; IGG, immunoglobulin G; OR, odds ratio; CI, confidence interval. Statistical significance level is indicated in bold font.

## 4 Discussion

This study highlights differences in urea, serum creatinine, and gamma GT at birth between foals that develop *R. equi* pneumonia and those that remain healthy. Interestingly, neonatal gamma GT has been identified as an early risk factor for the future development of the infection.

Our research was performed on foals less than 24 h of life. It has been proposed that lung infection is the result of inhalation of virulent *R. equi* by aerosols from contaminated soil (34), with intestinal colonization observed within the first week of life and *R. equi* being isolated in the feces of 3-day-old foals (35). Also, increased mRNA expression of different interleukins has been found in bronchoalveolar macrophages in foals 1 to 3 days old (9). This background justifies that the collection of samples was performed during the first day after the delivery, since it is suggested that the most likely time of infection would be soon after birth (22).

Urea is a product generated in the liver because of protein metabolism. The lower values observed in the foals that developed the disease in this study can also be explained by hepatic immaturity. Another possibility is that these foals had a diet lower in protein due to poorer milk quality from their mothers. However, this seems unlikely since there were no differences in colostrum quality, and the samples were taken within the first 24 h of life, which is not enough time for metabolic alterations attributable to diet to develop. In human neonates, changes in urea concentrations are due to factors other than dietary and renal ones (36). Interestingly, in human medicine, it has also been observed that premature neonates with functional immaturity are unable to increase plasma urea concentrations despite the administration of protein-rich diets, assuming that this is due to hepatic functional immaturity (37). This fact could explain why foals suffering from the disease had lower urea concentrations than healthy ones and could also be related to the immaturity of other systems, in this case, the respiratory immune system.

In this study, serum creatinine was higher in foals that developed *R. equi*. However, the mean value observed in those animals was within the range considered physiological in the neonatal foal (1.5–2.9 mg/dL) (38). Foals present a higher upper limit value than adult horses (39). Similarly, the serum concentration of gamma GT was higher in the sick group but also within the reference range. In neonatal foals, the reference ranges for hematological and biochemical parameters are highly variable and difficult to interpret. As both hypercreatinemia and pathological increased gamma GT levels have been linked to placental insufficiency, fetal stress, and immaturity (40, 41), is it likely that a certain level of immaturity might be present in foals that developed pneumonia in this study. Additionally, the importance of the adaptive immune system in human neonates has been highlighted in the development of respiratory diseases (42, 43). The association of gamma GT as a risk variable for *R. equi* pneumonia in this study could be considered a potential marker useful for identifying foals at risk, allowing for the necessary preventive measures to be taken to avoid the development of the infection.

Virulent *R. equi* replicates within macrophages, beginning its replication within 6–12 h, affecting macrophage viability within 48 h (3). Although macrophages do not prevent intracellular bacterial replication, *R. equi* infection induces an immune response in macrophages characterized by cytokine secretion (44). Studies in rats, pigs, and primates have shown that alveolar macrophages from young animals are less efficient, both in phagocytosis and in their ability to kill bacteria, particularly in the first weeks of life (45–47). Considering these findings and the research in horses, alveolar macrophages in newborn foals would be less bactericidal than in older foals (2). In our research, sick foals showed higher creatinine and gamma GT levels, indicating a certain degree of immaturity, which may induce a greater disease susceptibility. Indeed, several cases of pneumonia and other infections caused by *R. equi* have been detected in people (48–50), and this pathogen is always associated with states of immunosuppression (51–53).

Although in previous studies, WBC count and fibrinogen concentration have proven to be influential parameters in identifying foals affected with *R. equi*, no significant differences between groups were observed in this study evaluating foals less than 24 h of life. Low leukocyte and segmented neutrophil counts have been considered an early indicator of this disease when blood samples were obtained from foals aged 1 and 4 weeks (8), as well as a value of more than 13.000 leukocytes/μL in foals aged 3–5 weeks of age showed a sensitivity and specificity of 95.2 and 61.2%, respectively, to detect foals infected with *R. equi* pneumonia (33). Similarly, foals aged 3 to 19 weeks have also been analyzed hematologically with an interval of 4 weeks, where a total leukocyte count ≥13,000/μL and neutrophil count ≥10,000/μL were found with a sensitivity of 59 and 50% and a specificity of 37 and 55%, respectively (34).

All the studies mentioned in the previous paragraph were carried out in older foals and the samples were measured several times. This fact contrasts with our study, where blood samples were analyzed once within the first 24 h of life, which probably represents a more immature immune system. It has been observed that neutrophils have a reduced phagocytic capacity during the first 3 weeks and a decreased bactericidal capacity during the first 3 months of life (39). Both chemotaxis and neutrophil phagocytosis are low at birth, increasing significantly after colostrum ingestion (54). This suggests that newborn foal phagocytic cells are functionally mature but with limited chemotactic and phagocytic functions. According to this, age could be an important factor in the variability of the leukocyte count, especially the neutrophil count, so these values could not be considered reliable indicators to predict *R. equi* pneumonia within the first hours of life. Evidence suggests that foal neutrophils undergo numerical expansion and functional maturation in response to microbial exposure and other environmental stimuli (19, 55, 56), a phenomenon similarly observed in other species. The absence of significant differences in neutrophil counts may reflect insufficient postnatal time for activation or adaptation to environmental cues, as samples were obtained within the first 24 h of life. Furthermore, it seems evident that respiratory tract macrophages are involved in the initiation and early stages of this disease’s development, while neutrophils would be involved at a later stage, in the development of pulmonary abscesses (24, 57).

Similar to WBC count, serum fibrinogen concentration at birth was similar between healthy foals and those who became ill. While fibrinogen concentrations of 400 mg/dL are often considered the physiological upper limit in adult horses, it appears that in foals, the physiological values of fibrinogen vary most dramatically over the first weeks of life (27). Like our findings, previous studies have shown no differences in this parameter between foals aged 1 and 4 weeks (8). Another study observed an increase in plasma fibrinogen concentrations early in the development of *R. equi* pneumonia. Additionally, in foals older than 3 weeks of life, the sensitivity and specificity of fibrinogen concentration (≥600 mg/dL) as a predictive value were 59 and 33%, respectively (32), which means relatively low-reliability values. However, a more recent study analyzed weekly blood concentrations of fibrinogen in foals from the first 12 h of life to the seventh week, where it did observe statistically significant differences between concentrations of healthy foals versus preclinically/subclinically infected foals, suggesting fibrinogen is most likely to have a predictive value between the first and fourth week of infection, regardless of age (27). This background could explain our results, since considering that the incubation period of *R. equi* is 3 days to 4 weeks, depending on the dose of infection, and that the response time of equine fibrinogen occurs on average within 24–72 h, it is very unlikely that significant differences will be observed between the two groups studied within the first 24 h of life.

Serum concentration of total plasma proteins and albumin did not differ between the two groups studied, which was expected, since, to the author’s knowledge, they have not been directly related to the development of pneumonia by *R. equi.* However, we included these parameters as a measurement of total proteins and albumin are routinely assessed in the evaluation of neonatal foals to assess the degree of dehydration and/or failure of passive transfer of immunity, together with other markers such as urine density and IgGs. Additionally, these parameters were taken into account along with the parameters of the physical examination to determine if the foals were healthy at birth.

In this research, iron serum concentration did not change between healthy and sick foals, and values remained within the reference ranges (262–488 μg/dL) (57). This parameter was included in the study as iron is used by *R. equi* for the intracellular multiplication (6), which occurs around the first 6–12 h in culture, reaching a fivefold increase of bacteria after 48 h (3, 58). Accordingly, several studies propose the occurrence of an early infection of this pathogen after birth (7, 9). Additionally, in sick neonatal foals, plasma iron concentration does not seem to be an early indicator of systemic inflammation (57) unlike in adult horses, in which a decrease in serum iron levels is shown in acute inflammation (59). Based on this, we suggest monitoring iron concentrations to assess their relationship with the onset of clinical signs.

It has been hypothesized that failure of passive transfer immunity would increase the risks of infections and that foals with fewer colostrum antibodies are more susceptible to *R. equi* infection (60). Nevertheless, this study shows no difference in colostrum Brix values that suckled foals, nor in serum IgG concentration between healthy foals and those with pneumonia. These results are consistent with previous studies (16, 38). All these results indicate that the protection provided by colostrum and IgG levels are not strongly associated with the development of *R. equi*, a disease that is likely induced by a multifactorial cause (61). Indeed, a limitation of this study was that we only recruited one blood sample per foal, therefore, there was not a follow-up from birth to the onset of clinical signs of pneumonia. However, we aimed to identify changes at birth that may predict future development of the disease, as most studies that look for early indicators of *R. equi* pneumonia are carried out in foals *older* than 1 week. Additionally, although the sample size was high (207 foals), the number of sick foals was low (25 foals) and may limit the results obtained. In addition, due to the retrospective nature of this study, the sample size varied for each parameter analyzed. This was mainly caused by missing data and inconsistencies in the parameters originally recorded. Such heterogeneity in sample sizes may affect the statistical power of certain analyses, particularly those with fewer measurements, such as the fibrinogen.

To the author’s knowledge, this work is the first to assess predictive markers of *R. equi* pneumonia in neonatal foals in less than 24 h. Our results show differences in urea, serum concentrations of creatinine, and gamma GT between foals that became ill and those that remained healthy, and remarkably, these values remained in range. Since these findings could be related to the immaturity of macrophages and a more diminished innate immune response, it would be interesting to conduct studies that sought to relate neonatal immaturity to the risk of suffering pneumonia by *R. equi* in the future. In addition, findings in this report may indicate that special attention should be paid to neonatal foals presenting changes in these three parameters, mainly in endemic farms for *R. equi*. Interestingly, neonatal gamma GT may serve as a possible risk factor for developing the infection.

## Data Availability

The original contributions presented in the study are included in the article/Supplementary material, further inquiries can be directed to the corresponding author.
