# Supplementary material for: Blood parameters in neonatal foal and colostrum quality as possible early markers for increased risk of developing Rhodococcus equi pneumonia
Source: Front Vet Sci. 2025 Aug 29;12:1654052. doi: 10.3389/fvets.2025.1654052 (PMC12426006; doi:10.3389/fvets.2025.1654052)
Supplement: Supplementary file 1 [file Table_1.docx]

**Supplementary table 1:** additional data. F: Female; M: Male; Aa: AngloArab. NSF: No Significant Findings

| FOAL | SEX | BREED | DATE OF BIRTH | BRIX (%) | IGG (mg/dL) | FIBRINOGEN (g/dL) |  |
| --- | --- | --- | --- | --- | --- | --- | --- |
| 1 | F | Aa | 24/04 | 26% | 1478 | 336 | 4 lung checks, 0.5 cm microabscesses in the right lung, Not medicated |
| 2 | M | Arab | 25/04 | >30% | 1584 | 403 | 25/05- Ultrasound examination: NSF  5/07- Right lung ok, left lung with abscess 0.5cm  12/07 – Right lung OK, left lung abscess of >2cm. Start with azithromycin + rifampicin medication  31/08 – abscess reduced to 0.5 cm  10/09 – Discharge |
| 3 | F | Arab | 26/04 | 25% | 974 | 340 | 8/06 – Ultrasound examination: NSF  12/06 – Left lung abscess of >3 cm, medicated azithromycin + rifampicin  10/08 – Discharge |
| 4 | F | Arab | 28/04 | 26% | 1269 | 479 | 05/29 – Ultrasound examination: NSF  5/07 – Left lung, hepatized cranial area, and 2 cm abscess, medicated with azithromycin + rifampicin.  31/08 – Discharge |
| 5 | M | Arab | 29/04 | 26% | 1106 | 304 | 6 revisions, abscesses (2) of 2 cm were observed in the left lung, but no medication was taken.  the 2/10 clean lungs |
| 6 | F | Arab | 30/04 | 30% | 3122 | 560 | 29/05 – Ultrasound examination: NSF  20/06 – Left lung: several abscesses of 2-3cm. medicated with azithromycin + rifampicin.  10/09 – Discharge |
| 7 | F | Arab | 30/04 | 23% | 2963 | 380 | 7/06 – Medial malleolar fracture of the tibia.  9/08 – abdominal breathing. lungs with abscesses of 1 cm, medicated azithromycin + rifampicin.  30/09 – Discharge |
| 8 | M | Arab | 30/04 | >30% | 1911 | 332 | 12/06 – Right lung abscess 2.5 cm, medicated with azithromycin + rifampicin.  25/07 – Discharge |
| 9 | F | Arab | 1/05 | 21% | 844 | 447 | 9/07 – Right lung, abscess 2 cm. left lung abscess 2-3cm, medicated with azithromycin + rifampicin.  2/10 – Discharge |
| 10 | M | Arab | 1/05 | 30% | 654 | 386 | 25/05 – Ultrasound examination: NSF  3/07 – Ultrasound examination: NSF  12/07 – Ultrasound examination: NSF  20/07 – Ultrasound examination: NSF  31/07 – Left lung: <1CM abscess, Right lung: microabscesses.  10/08 – Right pleural effusion + deep flat abscess, medicated with azithromycin + rifampicin.  22/10 – Discharge |
| 11 | F | Arab | 1/05 | 21% | 1289 | 502 | 12/06 – Ultrasound examination: NSF  10/07 – Superficial corneal ulcer. Right lung: microabscesses.  13/08 – Right lung abscess 2x3 cm, medicated with azithromycin + rifampicin.  22/10 – Discharge |
| 12 | M | Arab | 2/05 | 26% | 1579 | 383 | 7 check-ups, some microabscesses but no medication. |
| 13 | M | Arab | 4/05 | >30% | 1584 | 288 | 14/06 – Ultrasound examination: NSF  10/07 – Ultrasound examination: NSF  26/07 – Right lung 0.5cm, left lung abscess 2x1cm.  13/08 – Both with many abscesses of 2 and 1 cm, azithromycin + rifampicin medication begins.  12/11 – Discharge |
| 14 | F | Arab | 7/05 | >30% | 2040 | 313 | 29/05 – Ultrasound examination: NSF  5/07 – Right lung in cranial area, abscess 3 cm and caudal 2 cm, medicated with azithromycin + rifampicin.  31/08 – Discharge |
| 15 | F | Arab | 9/05 | >30% | 2554 | 289 | 12/06 – Ultrasound examination: NSF  19/07 – Ultrasound examination: NSF  20/08 – Right lung: several abscesses of 1 and 2 cm; Left lung in cranial abscess 1.5 cm, medicated with azithromycin + rifampicin.  12/11 – Discharge |
| 16 | F | Arab | 10/05 | 24% | 470 | - | 12/06 – Ultrasound examination: NSF  16/08 – Ultrasound examination: NSF  30/08 – Both lungs with pleurisy. Medicated with azithromycin + rifampicin.  12/11 – Discharge |
| 17 | M | Aa | 10/05 | 24% | 1246 | 497 | 8/06 – Ultrasound examination: NSF  5/07 – Microabscesses in both lungs, medicated with azithromycin + rifampicin.  31/08 – Discharge |
| 18 | M | Arab | 16/05 | >30% | 1365 | - | 14/06 – Ultrasound examination: NSF  10/07 – Pleurisy of both lungs. Medicated azithromycin + rifampicin.  10/09 – Discharge |
| 19 | M | Arab | 17/05 | 30% | 2068 | 322 | 9/07 – Lungs with microabscesses (<1cm).  07/19 – Microabscesses all over the surface.  07/26 – They increase in size >1cm, medicated with azithromycin + rifampicin.  12/11 – Discharge |
| 20 | F | Aa | 21/05 | >30% | 1091 | 353 | 20/07 – Ultrasound examination: NSF  6/08 – Abscesses of 1-2cm from both lungs, medicated azithromycin + rifampicin.  22/10 – Discharge |
| 21 | M | Arab | 21/05 | 22% | 1069 | 356 | 05/21 – Omphalitis, treatment with sulfonamides.  26/07 – Left lung abscess 1 cm, medicated with azithromycin + rifampicin.  22/10 – Discharge |
| 22 | F | Aa | 24/05 | 20% | 984 | - | 10/07 – Fever and nasal discharge. Ultrasound: Comet tails in a band through both lungs.  11/07 – Dead |
| 23 | F | Arab | 26/05 | - | 1170 | 566 | 9/07 – Left lung: cranial abscess 3x2 cm. Right lung: hepatized cranial area. Treatment with azithromycin + rifampicin.  07/20 – Septic arthritis of the tarsus, abdominal bracing, changed to clarithromycin + rifampicin.  07/25 – Dead |
| 24 | F | Arab | 3/06 | >30% | 1794 | 378 | 9/07 – Right lung: abscesses of >1cm in cranial, middle and caudal. Left lung: NSF. Medicated with azithromycin + rifampicin.  2/10 – Discharge |
| 25 | F | Arab | 3/06 | 25% | 1405 | 413 | 9/07 – Pleurisy in both lungs, medicated with azithromycin + rifampicin.  16/07 - Discharge |
